# A Geographic Weighted Regression Analysis of the Health Opportunity Index and Stroke Prevalence in Health and Human Services Region 3

**DOI:** 10.3390/ijerph22101542

**Published:** 2025-10-09

**Authors:** Wanderimam R. Tuktur, Bin Cai, Howell C. Sasser, Rexford Anson-Dwamena

**Affiliations:** 1 Office of Health Equity, District of Columbia Department of Health, 2201 Shannon Pl SE, Washington, DC 20020, USA; 2 College of Health Sciences & Public Policy, Walden University, 100 Washington Avenue South, Suite 900, Minneapolis, MN 55401, USA; 3 Office of Health Equity, Virginia Department of Health, Richmond, VA 23219, USA; rexford.dwamena@vdh.virginia.gov

**Keywords:** Health Opportunity Index (HOI), stroke prevalence, geospatial, social determinants of health, Health and Human Services Region 3 (HHS Region 3), health disparities, neighborhood effect, population study, geographic weighted regression, public health

## Abstract

Although stroke prevalence remains one of the leading causes of death and morbidity in the United States, there is paucity of ecological studies at the census tract level that elucidate geospatial associations between predictors of stroke prevalence in states across U.S. Health and Human Services Region 3 (HHS Region 3: Delaware, Maryland, Pennsylvania, Virginia, West Virginia, and the District of Columbia). This study operationalized the Health Opportunity Index (HOI) by exploring the geospatial relationship between the 13 indicators of the HOI and stroke prevalence at the census tract level in HHS Region 3 using four HOI indicator profiles: (a) neighborhood and built environment profile, (b) social and community context profile, (c) resource profile, and (d) economic profile. The methodological approach was quantitative using secondary data. The sample size was 8021 census tracts. The HOI was estimated for each census tract in the study area. Geographic weighted regression model was run to examine the varying strengths and direction of geospatial relationship of 13 HOI indicators and stroke prevalence across census tracts in HHS Region 3. The results showed variation in the geographic weighted regression (GWR) local estimated coefficients for each indicator across the study area, reflecting variation in the strength and direction of the associations. The findings of our study can guide the identification of geographic priorities for resource allocation, design of quality improvement interventions, inform policy creation and targeted local strategies for stroke prevention services across neighborhoods, support grant applications, and inform future research on stroke prevalence in HHS Region 3.

## 1. Background

Stroke is an acute neurological disorder that leads to hospital admissions [1]. Globally, stroke is the second leading cause of death [2,3,4]. According to the Global Burden of Disease Stroke Collaborators, annual stroke cases and deaths rose significantly worldwide between 1990 and 2019 [5].

While stroke remained the fifth leading cause of death in the U.S. from 2016 to 2018, its prevalence and incidence among people under 70 grew by 22% and 15%, respectively [6,7]. A county-level analysis of stroke mortality in the United States showed that despite stagnation in the national decline of stroke death rates, there is significant geographic variation [6]. The study found that while 40% of counties saw a 20% reduction in stroke mortality, other counties experienced an increase in stroke burden [6]. Disparities were observed by age group, race/ethnicity, and geography, with increases in stroke risk factors and hospitalizations noted especially among younger adults and in less urban and rural counties [6].

There are documented disparities in stroke prevalence and mortality. While stroke risk generally increases with age, with the highest disparities observed in young adults, these disparities lessen significantly after age 65 [8]. These inequities are partly linked to higher prevalence of hypertension, diabetes, and other risk factors in minority populations, compounded by SDOH, such as inadequate education, income, and limited healthcare access [8,9]. Understanding factors that account for these disparities in stroke prevalence is critical for accelerating equitable stroke prevention.

Significant racial and ethnic inequities exist in stroke prevalence, with a greater burden on African Americans than any other racial/ethnic group in the United States [10]. The authors highlighted multiple negative stroke outcomes for ethnic minorities, including significantly higher incidence (more than two-fold), recurrence, and mortality rates, as well as inequities in stroke knowledge and care [10]. Robertson and colleagues found that non-Hispanic Black adults, individuals with less than a high school education, and those in the lowest income bracket experienced the highest stroke prevalence [11]. As racial and ethnic minorities are projected to make up 40% of the U.S. population by 2030, addressing disparities in stroke prevalence is a growing necessity [12].

A study highlighted significant disparities in stroke mortality, reporting 1.22 times higher odds of death within 30 days of a stroke for patients in large rural cities and towns compared to urban residents [13]. These rural-urban discrepancies extend to stroke incidence, risk factors, case fatality, and stroke care [13]. Nevertheless, rural stroke death rates, though still higher than urban rates in 2017 (by 8%), showed a significant improvement from 2007, when the disparity was 16% [14].

To effectively address the root causes of stroke, both clinical interventions and a focus on social determinants of health (SDOH) is necessary [6].

This approach is informed by studies on stroke incidence, prevalence, mortality, disability, risk factors, and epidemiological trends, and is vital for evidence-based care, planning, and resource allocation.

Subsequent sections of the background highlight the neighborhood effect of SDOH, the HOI, research problem, study justification and purpose.

### 1.1. Neighborhood Effect of Social Determinants of Health

In the context of social epidemiology and health, the *neighborhood effect* is defined as the independent causal effect of residential community on any number of health and/or social outcomes [15].

Studies have demonstrated the relationship between neighborhood deprivation and stroke mortality [16]. For example, a study analyzed stroke mortality data using census block groups as a proxy for neighborhoods in the state of Arkansas [17]. They found that poverty and education drive disparity in stroke mortality rates, particularly in socioeconomically disadvantaged neighborhoods [17]. Geographically Weighted Regression (GWR) showed these factors, along with population density and mobility, explained 4.5% to 9% of the variation in stroke mortality, emphasizing the need to address neighborhood-level socioeconomic disparities in public health interventions [17].

### 1.2. Health Opportunity Index (HOI): A Tool for Measuring SDOH

Despite been a relatively new tool in public health, the HOI efficiently demonstrates the *neighborhood effect* of SDOH on health outcomes by using specific variables to effectively show how a neighborhood’s social conditions influence health. This tool is similar to the more established Child Opportunity Index (COI) and facilitates the comparison of *health opportunity* levels across different neighborhoods [18].

The HOI provides a framework for comprehending the pervasive influence of SDOH on stroke prevalence disparities. By operationalizing these factors as area-level conditions, distinct from individual-level SDOH, the HOI facilitates analysis of how these upstream systemic factors interact to perpetuate unequal health outcomes.

Environmental indicators, including walkability and environmental quality, are captured by the HOI and are crucial SDOH. The natural and built environments, encompassing everything from climate change to green spaces and community areas, directly affect health outcomes [19]. For example, studies show that exposure to toxins and pollution adversely affect mental health [19], whereas access to urban green spaces, such as urban parks are linked to the prevention of cardiovascular diseases such as stroke [20]. However, Nunez and colleagues caution that confounding variables, like the proliferation of fast-food restaurants, can mediate the relationship between green space and cardiometabolic health, potentially serving as a stronger predictor of obesity, a prelude for stroke [21]. Additionally, characteristics of the built environment, including elements like buildings, sidewalks, and roads, as well as accessibility, aesthetics, and neighborhood conditions, are a reflection of social, political, and economic processes and priorities [19].

The HOI captures other key SDOH including access to healthcare, education, and employment. Studies show that the availability of preventive care and therapeutics, influenced by policy and funding, can affect individual health and lead to health inequities [19]. Education is another important factor, as more educated individuals generally experience better health, and their educational level is often related to their employment access [19].

Housing and transportation costs are critical factors in the HOI’s affordability index, which influence health outcomes. Housing instability, insecurity, and homelessness lead to health disparities [19]. Similarly, transportation plays an important role as a SDOH. Access to reliable transportation is essential to access necessities like education, employment, healthcare, and healthy food. These interconnected variables are measured and accounted for through various indicators within the HOI framework.

The HOI measures several factors crucial to economic growth and income, including job participation, food access, and residential segregation [18]. Each of these factors has significant health implications. For example, residential segregation is a root cause of health inequities, while limited access to healthy food, particularly in “food deserts,” directly leads to poor nutrition and reduced physical activity [19].

### 1.3. Research Problem

The research problem is the persistent disparities in stroke prevalence and outcomes across the United State and the scarcity of ecological research that explores how specific neighborhood-level predictors of health, quantified by indices of the HOI, are geospatially associated with stroke prevalence at a localized, census tract level within HHS Region 3: Delaware, Maryland, Pennsylvania, Virginia, West Virginia, and the District of Columbia.

The study is warranted to prioritize the most at-need census tracts, particularly related to neighborhood-level SDOH that account for disparities in stroke prevalence, for better control of stroke risk factors, and to inform concerted local strategies and planning of stroke prevention services across neighborhoods in HHS Region 3.

### 1.4. Study Justification/Purpose

Generally, researchers have reported on the paucity of published population-level studies that associate stroke hospitalization and mortality with a large set of SDOH [9]. Numerous multilevel modeling studies have explored how individual characteristics and social context influence an individual’s health but have yielded inconsistent results [22]. Additionally, studies on individual factors alone do not provide a holistic picture of the *place effect*, which includes social, economic, and physical conditions in the environment where people live, i.e., SDOH [23]. Furthermore, capitalizing on the contributors of health outcomes (clinical care, health behaviors, socioeconomic factors, and physical environment) by individual factors alone will detract from adopting policies or implementing interventions with greater precision for target populations [23].

Existing indices of deprivation or opportunity often operate at the county or state level, obscuring more granular disparities. A clear example is Fairfax County, Virginia, where the county-wide poverty rate is 6%, yet specific census tracts exhibit much higher rates of 18.6% and 13.7% [24]. Our application of the HOI at the census tract level closes this critical gap by providing a more detailed analysis.

Since the percentage of racial and ethnic minorities in the U.S. is projected to almost double by 2050, there is an increasing urgency to reduce racial-ethnic disparities in health outcomes [25]. Our geospatial study directly addresses this need by investigating how geographic variations in stroke prevalence in HHS Region 3 reflect these persistent inequities, particularly among ethnic minorities.

Furthermore, the use of advanced tools like geographic information systems (GIS) and multilevel modeling has enhanced our ability to examine SDOH at the neighborhood level [23]. Research shows that comprehensive deprivation indices are more effective for measuring broad socioeconomic differences than are specific measures of education or wealth [23].

By using a geospatial approach to analyze the relationship between the neighborhood context (HOI indices) and stroke prevalence in HHS Region 3, we can provide public health planners with crucial information to strategically allocate specific facilities and services to the communities most in need of stroke prevention measures.

HHS Region 3 is known for its remarkable diversity with a stretch of sprawling suburbs, rural communities, and urban cities [26]. However, health disparities in states within HHS Region 3 have driven attention toward focusing on health equity, bridging the urban–rural divide, and improving the health of residents of HHS Region 3 [26].

Therefore, the purpose of this study is to examine the geospatial relationship between the 13 indicators of the HOI (categorized into four profiles) and stroke prevalence in HHS Region 3, which consists of five states and one district: Maryland, Pennsylvania, Virginia, West Virginia, Delaware, and the District of Columbia. The aim of our study is to examine the varying directionality and strengths of association between the 13 indicators of the HOI and stroke prevalence across census tracts in HHS Region 3.

We begin by providing a summary of the construction of the HOI, first created by Rexford Anson Dwamena, an epidemiologist in Virginia Department of Health and applied to small geographies nationally. We then incorporate the HOI into GWR models to determine the direction and strengths of association between the 13 HOI indicators and stroke prevalence across census tracts in HHS Region 3. Finally, we provide interpretation for each HOI indicator GWR coefficient map and provide policy implications of the results.

## 2. Methodology

We adopted an ecological study design and quantitative research methodology for this study. The independent variables are the 13 indicators of the HOI: education, income inequality, Townsend deprivation, job participation, healthcare access, walkability, food access, environmental quality, geographic mobility, population weighted density, segregation, employment access, and affordability indices [18]. The dependent variable is stroke prevalence. Sub-sections of the methodology include data sources, HOI computation, and data analysis.

### 2.1. Data Sources

Secondary datasets were utilized for the study. Small area estimates (census tracts) derived from Behavioral Risk Factor Surveillance System (BRFSS) survey for *population 18 years and above* was the data source for stroke prevalence and obtained from CDC PLACES datasets for the year 2020. The 13 HOI indicators were computed from data variables obtained from the following United States federal agencies:

(a) U.S Census Bureau, American Community Survey (ACS) datasets: Education index, income inequality, Job participation, Townsend deprivation index, Spatial segregation index, Population churning, Population weighted density
(b) USDA Food Access Research Atlas: Food Access Index
(c) Center for Neighborhood Technology (CNT) Datasets: Affordability Index, Employment Access Index
(d) U.S. Environmental Protection Agency (EPA), Environmental Justice Screening (EJScreen) datasets: Environmental Quality Index, Walkability Index
(e) Health Resources and Services Administration (HRSA): Healthcare Access Index

A population-weighted method was employed to transform stroke prevalence data, which was based on the old 2010 census tract boundaries [27]. This was necessary because data for computing the Health Opportunity Index (HOI) indicators were collected at the new 2020 census tract level [27].

Initial data screening was performed on 8046 census tracts from HHS Region 3. A preliminary assessment revealed no missing data. We identified and removed 25 census tracts that fell more than three standard deviations from the mean on scatter plots and normal distribution curves of stroke prevalence data. The final analysis consisted of 8021 census tracts, representing a 0.3% reduction from the original dataset.

### 2.2. HOI Computation

Nonspatial analysis was used to calculate the HOI composite score for each census tract in HHS Region 3’s five states and district. This computation was performed after first creating 13 distinct indices from 2022 datasets obtained from U.S. federal agencies [18] (outlined above).

We normalized 13 HOI indices for each census tract across HHS Region 3 (five states and one district) by calculating non-geospatial z-scores. Z scores were estimated by calculating the difference between the census tract value for a given indicator and the average value across all census tracts in a state and then dividing this difference by the standard deviation for all census tracts in the given state [28]. Using the formula:

*z* = (raw value − mean)/standard deviation (1)

For negatively oriented indicators, where higher values correspond to adverse outcomes, the values were reverse-coded prior to analysis. Consequently, in interpreting each indicator, high absolute values for each indicator represent a desirable outcome (e.g., high income inequality value implies low-income gap). Conversely, low indicator values connote an undesirable outcome.

The *z*-scores of all 13 indices computed were subjected to a non-geographical dependent principal component analysis (PCA) using SPSS statistical software (version 28). PCA is a data reduction technique. PCA reduced the 13 indicators into fewer linear combinations (called *profiles*) based on indicators most closely associated with one another. The four HOI profiles are as follows: (a) neighborhood and built environment profile (affordability, walkability, employment access, and population-weighted density indices); (b) social and community context profile (job participation, education, food access, Townsend material deprivation, and geographic mobility indicators); (c) resource profile (healthcare access and spatial segregation indices); and (d) economic profile (income inequality and environmental hazard indicators). Weights for each profile were generated, and all four weighted profiles were summed across each census tract to generate a HOI composite score per census tract. HOI scores for each census tract ranged from 0 to 1. The closer the score is to zero, the lower the probability that individuals in the census tract have opportunities to achieve optimal health. Conversely, a score closer to 1 signifies greater opportunity to achieve good health.

### 2.3. Data Analysis Plan

We ran a GWR model using the 13 computed HOI indices. GWR has increasingly been utilized to study spatially varied relationships over a geographic area [15]. GWR was utilized to model spatially varying associations, such as accounting for spatial variability in stroke prevalence across census tracts in the study area [29].

GWR utilizes predictors and dependent variables within the neighborhood of a target census tract to construct a local linear regression model for prediction and interpretation [30]. GWR allowed a different regression model at each spatial location (e.g., each census tract) and the regression coefficient changed across census tracts within our study area [30]. Hence, the predictors had different impacts on the dependent variable at different census tracts in the study area. GWR achieved this by creating a weighted regression model for each census tract using the HOI indices and stroke prevalence of the census tract and its spatial neighbors. This approach is informed by the first law of geography that states that things that are close together are more related than distant features. Hence, census tracts closer to a target census tract have a larger influence on the local regression model and are assigned higher weights [30].

MGWR (multiscale geographic weighted regression) is an extension of GWR. Although, GWR utilizes predictors and dependent variables within the neighborhood of a target census tract to construct a local linear regression model for prediction and interpretation [31]. MGWR employs an advanced spatial regression technique [31]. Hence, our analysis used MGWR which provides more accurate local regression coefficients. However, for consistency with existing literature and to prevent ambiguity, GWR is used throughout this paper.

Following the GWR analysis, the local coefficients were mapped to visualize the spatial heterogeneity in the strength and direction of the associations between the 13 indicators of the HOI and stroke prevalence by census tract in the study area.

## 3. Results

### 3.1. Demographic Characteristics of HHS Region 3

Table 1 shows that three HHS Region 3 states—West Virginia (21.5%), Delaware (21.3%), and Pennsylvania (20.0%)—exceeded the national average of 17.7% for people aged 65 and older. In contrast, the District of Columbia had the lowest proportion at 13.1%. Consequently, because those 65 and older are Medicare-eligible, the percentage of uninsured individuals in all five states and the district is lower than the national average [24].

The proportion of non-Hispanic Whites was highest in the state of West Virginia (90.9%) and Pennsylvania (74.1%). Similarly, the highest proportion of Blacks was reported in Maryland (31.6%) and the District of Columbia (44.4%) [24].

HHS Region 3 consistently shows high rates of educational attainment (high school graduates or higher) across all five states and the district. Job participation for the population ages 16 and over was greatest in District of Columbia (71.4%) and lowest in West Virginia (53.1%). Median household income was lowest in West Virginia ($55,217) and Pennsylvania ($73,170) and highest in the District of Columbia ($101,722) and Maryland ($98,461), with a national average of $75,149. Paradoxically, persons living in poverty was highest in the District of Columbia (13.30%) and lowest in Delaware (9.4%), with a national average of 11.1% [24].

### 3.2. Interpretation of GWR Coefficient Maps

The GWR local estimated coefficients represents the strength and direction of association between a given HOI indicator and stroke prevalence across census tracts in HHS Region 3.

The t-statistic in GWR is the “adjusted critical value used to test the statistical significance of the GWR coefficients in a two-sided *t*-test at 95% confidence” [30]. The t-statistics in GWR measures how many standard errors the estimated GWR coefficient is from zero [30]. The larger the absolute value of the t-statistic, the more significant and reliable the estimated GWR coefficient, and connotes the coefficient is unlikely to be zero, a conclusion reinforced by a low *p*-value [30].

Both parameters (GWR coefficient and t-statistic) are mapped for each explanatory variable (HOI indicator) in the appendix. In each map, positive local coefficients (representing positive strengths of association between the HOI indicator and stroke prevalence across census tracts in the study area) were colored green. Negative local coefficients (representing negative strengths of association between the HOI indicator and stroke prevalence across census tracts in the study area) were colored red.

Additionally, the background of the map was shaded either deep ash, lighter ash or white and represents census tracts across the study area where the local parameter estimates were significant at the 0.05 alpha level (deep ash), 0.1 alpha level (light ash), or non-significant (white).

#### 3.2.1. Neighborhood and Built Environment (Profile1)

Table 2 shows the GWR model diagnostics for neighborhood and built environment profile (Profile 1). The proportion of the variance of stroke prevalence accounted for by profile 1 GWR model was (*R*^2^ = 76.90%) [30]. Profile 1 was the second-best performing model with an AICc (=15,537.40). AICc is a measure of model performance, and lower AICc values provide a better fit to the observed data [30]. Unlike Ordinary Least Square (OLS) regression models where the prediction is based on a global model, in GWR, the prediction is based on a local model that is calibrated using neighboring census tracts

#### 3.2.2. Employment Access Indicator

Appendix A shows employment access indicator with negative coefficients dispersed widely across the District of Columbia. Majority of the tracts were statistically significant at 0.05 alpha level and implied that lower employment access in DC, particularly around Wards 5, 7, and 8, was associated with high stroke prevalence in those areas. Negative coefficients were equally seen in the Appalachia area of West Virginia. However, an opposite spatial pattern was observed in census tracts within Kent County and Delaware, where positive coefficients are seen.

#### 3.2.3. Affordability Indicator

Appendix B shows affordability indicator with statistically significant negative coefficients in census tracts within southside Virginia. Hence, as affordability (a function of housing cost and transportation as a proportion of earned income) decreased, stroke prevalence increased. Similarly, statistically significant negative coefficients were seen in some census tracts in Union County of Pennsylvania. On the contrary, positive coefficients were seen in census tracts within the northern part of West Virginia that shares a border with Pennsylvania. The higher the affordability in those areas, the higher the stroke prevalence. A similar positive pattern was seen within the central and southern parts of DC. The tracts described and interpreted were statistically significant.

#### 3.2.4. Walkability Indicator

Appendix C highlights walkability indicator GWR coefficient map. Most tracts over Baltimore city in the state of Maryland showed statistically significant negative coefficients. Hence, as walkability decreased, stroke prevalence increased. Similarly, census tracts across the lower belt of Virginia that share a border with North Carolina had negative coefficients. Conversely, statistically significant positive coefficients were seen in census tracts in Ward 4 and 8 of DC.

#### 3.2.5. Population Weighted Density Indicator

Appendix D shows PWD indicator GWR maps and census tracts with negative coefficients across the Appalachia region of Virginia, West Virginia, and Pennsylvania. Hence, as PWD decreased, stroke prevalence increased. A similar finding was seen in Frederick (Maryland) and Baltimore.

#### 3.2.6. Social and Community Context (Profile 2)

Table 3 shows the GWR model diagnostics for social and community context profile (Profile 2). The proportion of the variance of stroke prevalence accounted for by the regression model was (*R*^2^ = 79.90%) [30]. Profile 2 model had the lowest AICc (=12,173.69), hence, it outperformed the other three models and provided the best fit to the observed data when compared to Profile 1, 3, and 4 models [30]. Additionally, Profile 2 had the lowest sigma-squared and sigma-squared MLE, which is supporting evidence of a better performing model, because smaller values of these statistics are preferred [30].

#### 3.2.7. Geographic Mobility Indicator

Appendix E shows the geographic mobility indicator and positive coefficients in the western part of Philadelphia. These statistically significant census tracts highlight more stable populations and are associated with higher stroke prevalence. Positive coefficients were also seen in census tracts around Centre County of Pennsylvania and the southern part of Virginia, such as Dinwiddie, Halifax, Prince Edward, Brunswick, and Campbell, which are all rural counties. The census tracts described were all statistically significant.

#### 3.2.8. Townsend Deprivation Indicator

Appendix F shows a wide coverage of statistically significant negative coefficients across the study area for Townsend deprivation index. Hence, as deprivation decreased, stroke prevalence decreased. However, there were a few areas with positive coefficients, which were not statistically significant.

#### 3.2.9. Food Access Indicator

Appendix G shows statistically significant negative coefficients for food access indicator across the study area, with the exception of a small region in the northern part of Pennsylvania, which was not statistically significant. Hence, areas with greater access to grocery stores (within 10 miles in rural areas and 1 mile in urban areas) had less stroke prevalence.

#### 3.2.10. Education Indicator

Appendix H highlights statistically significant negative coefficients for the educational attainment indicator in all census tracts across the study area.

#### 3.2.11. Job Participation Indicator

Appendix I shows negative coefficients in most tracts across the study area. Hence, an increase in job participation was associated with a decrease in stroke prevalence. However, some other tracts showed positive coefficients, such as those around Centre County, of Pennsylvania. On subsequent scrutiny of census tracts around Centre County using bivariate maps, the positive coefficients observed in those census tracts were driven by low job participation and associated with low stroke prevalence.

#### 3.2.12. Resource Profile (Profile 3)

Table 4 shows the GWR model diagnostics for resource profile (Profile 3). The proportion of the variance of stroke prevalence accounted for by the regression model was (*R*^2^ = 68.24%) [30].

#### 3.2.13. Segregation Indicator

Appendix J highlights the segregation indicator with negative coefficients in census tracts across Allegheny County, in the western part of Pennsylvania. Hence, as diversity in an area increased, stroke prevalence decreased

#### 3.2.14. Health Access Indicator

Appendix K shows Access to healthcare indicator. Positive coefficients are seen across census tracts in DC, Baltimore, and Philadelphia. On subsequent analysis using bivariate maps, DC showed high healthcare access and high stroke prevalence in pockets across wards 4, 5, 7, and 8.

#### 3.2.15. Economic Profile (Profile 4)

Table 5 shows the GWR model diagnostics for economic profile (Profile 4). The proportion of the variance of stroke prevalence accounted for by the regression model was (*R*^2^ = 69.91%) [30]. Profile 4 model was the least performing model (highest AICc = 15,988.29) compared to all other models.

#### 3.2.16. Income Inequality Indicator

Appendix L shows the income inequality indicator with statistically significant negative coefficients across census tracts in DC. On subsequent analysis using bivariate maps, the negative coefficients in DC represent low-income inequality (wide income gap) associated with increased stroke prevalence.

Conversely, positive coefficients in some census tracts in Prince George’s County that share a border with DC connote a narrow income gap, which was associated with increased stroke prevalence.

#### 3.2.17. Environmental Hazard Indicator

Appendix M shows GWR coefficient maps for environmental hazard indicator, which highlights negative coefficients around Norfolk, Portsmouth, and Danville areas in Virginia. Hence, as environmental pollution increased, stroke prevalence increased. Conversely, positive coefficients were seen in census tracts around the city of Philadelphia.

### 3.3. GWR Residual Diagnostics

The spatially random distribution of the results from GWR models 3 and 4 suggests that the two models satisfy the “no spatial autocorrelation” assumption for the residuals (see Supplementary File S1). Consequently, the errors are not influenced by the errors of the neighboring census tracts. Additionally, it indicates that the errors of these two models are normally distributed and have a constant variance [32]. Nevertheless, GWR models 1 and 2 exhibit spatial dispersion that suggests high residuals are surrounded by low residuals, or vice versa [32]. The Moran’s I result contradicts the model’s assumption that the residuals have a normal distribution, are independent of the residual, and have a constant variance. Nevertheless, the residuals from these two models appear to be normally distributed in the histogram (see Supplementary File S1).

## 4. Discussion

Our study found variation in the direction and strength of association between indicators of the HOI and stroke prevalence across census tracts in HHS Region 3. For example, employment access indicator had statistically significant negative coefficients in the Appalachia area of West Virginia and widely across District of Columbia, but an opposite spatial pattern of positive coefficients was seen in Delaware.

According to our GWR analysis, lower access to employment is associated with high stroke prevalence in Washington, D.C., particularly in Wards 5, 7, and 8. Key determinants of employment access, such as proximity to employment centers and transportation availability are potential drivers of this association and initiate a cascade of adverse socioeconomic effects, including employment instability and greater vulnerability [18], which are known precursors to stroke. The effect is compounded for individuals with limited employment readiness, who face compounded systemic challenges involving transportation, housing, child-care, and insufficient public transit in lower-income communities [33]. As evidence of this socioeconomically driven disparity, D.C. carries a poverty rate of 13.30%, higher than the national average (11.10%) and all other states and districts within HHS Region 3.

As demonstrated by Hoehner and colleagues, the link between employment access and stroke prevalence is an important consideration [34]. Their study found that increased commuting distance was associated with lower physical activity and cardiorespiratory fitness, higher BMI, and elevated blood pressure [34]. In this context, extended commutes can trigger a cycle of declining health, increasing the risk for obesity and hypertension, which are established stroke precursors [35].

The GWR coefficient map for our walkability indicator demonstrated that a decrease in walkability in cities like Baltimore (with walkable neighborhoods) is associated with a rise in stroke prevalence. This finding may be explained by social factors, including neighborhood safety and pedestrian fatalities, which can discourage walking, thus contributes to the higher stroke prevalence. In Baltimore, for example, pedestrian fatalities account for 30–47% of all fatal crashes [36].

While increased walkability is typically associated with better health, our analysis revealed the opposite in census tracts within D.C Wards 4 and 8, where higher walkability was associated with increased stroke prevalence. We contend that this counterintuitive finding is not a causal relationship but rather a reflection of confounding variables rooted in the historical social and structural disadvantages prevalent in these areas, as well as in Wards 5 and 7. The D.C. Policy Center has previously highlighted that Washington D.C.’s underserved communities face higher rates of obesity and inactivity, get less than seven hours of sleep per night, and have fewer walkable areas [37]. These historical long-standing health disparities are likely the primary drivers of the increased stroke prevalence observed in our study, with the rise in walkability occurring in communities already at higher risk for stroke.

Findings from multiple studies highlight the health benefits of a walkable built environment and an active lifestyle. Some researchers developed a neighborhood walkability index (NWI) and found that participants living in areas with higher NWI, over time, had lower BMI and waist circumference [38]. The study concluded that residential neighborhood features supporting pedestrian activity were associated with lower adiposity [38]. This research supports broader findings that link lifestyle factors to significant health outcomes. For example, a study identified that 74.2% of variable risk factors for stroke are associated with a lack of physical exercise, poor diet, and other lifestyle choices [39]. The importance of physical activity is further highlighted by studies that reported that regular exercise is vital for decreasing the incidence of stroke [39,40].

In the Appalachian areas of Virginia, West Virginia, and Pennsylvania, our GWR coefficient map showed that low population weighted density (PWD) was associated with increased stroke prevalence. This finding is further contextualized by significant demographic and systemic factors, particularly in West Virginia census tracts. These areas are rural, with less dense population per square mile, higher stroke prevalence and overlap with healthcare professional shortage areas [41]. West Virginia is entirely situated within Appalachia and possesses the nation’s third-largest population of individuals aged 65 and older—a demographic with greater chronic health needs and heightened stroke risk [41]. The complex interplay of aging population, rurality, healthcare access issues, mistrust of healthcare systems, and historical health and economic inequities within Appalachia further exacerbate these health challenges, as noted in this study [41].

Published articles corroborate our results, with research consistently demonstrating rural-urban disparities in stroke risk. A study reported that stroke risk factors such as hypertension, obesity, smoking, and physical inactivity are more common in rural populations, which also have lower access to healthcare providers [42]. Another study found that rural residents have lower income and education, lower socioeconomic status, greater transportation issues, less access to healthy food, greater adverse levels of psychosocial factors, and increased exposure to environmental contaminants, all of which are linked to a higher incidence of stroke [13].

The spatial patterns of the GWR coefficient for affordability indicator showed significant regional variation. In some areas, such as census tracts in southside Virginia and Union County, Pennsylvania, the expected negative relationship was observed: decreasing affordability was associated with an increase in stroke prevalence. This link is likely due to reduced disposable income, which curtails residents’ access to healthcare and adequate nutrition. However, this relationship was inverted in other locations. Paradoxically, higher affordability was associated with higher stroke prevalence in northern West Virginia and the central and southern parts of D.C. For example, despite lower average rent in D.C.’s Wards 7 and 8 (60% less) compared to Wards 2 and 3 [43], these areas exhibit higher stroke prevalence. This seemingly contradictory finding in D.C is likely driven by underlying social and structural inequities. Residents of these more affordable wards (wards 7 and 8), experience higher poverty, lower high school graduation rates and are home to majority of the city’s food deserts, which contribute to higher obesity rates and related chronic conditions like stroke [44].

In HHS Region 3, an inverse relationship was observed between educational attainment and stroke prevalence, as shown by the GWR coefficient map. This finding aligns with another study which reported that higher educational attainment correlates with more health knowledge and a greater willingness to engage in physical activity, thereby reducing stroke-related comorbidities [20]. Similarly, Zhang and colleagues found that among rural patients with acute ischemic stroke, higher educational attainment was associated with a 24.6 times lower rate of decision-making delays [45]. The authors explained that greater stroke knowledge among educated individuals helps in the early identification of symptoms and warning signs [45].

Geographic mobility, also known as population churning, is a standardized metric of population movement, defined as the “combined inflow (in-migration) and outflow (out-migration) of people from a census tract as a proportion of the total population of the given census tract” [18]. GWR coefficient map demonstrated that in western Philadelphia, southern Virginia, and census tracts around Centre County, Pennsylvania, increased population stability—measured as an increase in the geographic mobility indicator was associated with higher stroke prevalence. 

A possible explanation for the observed association in western Philadelphia is the presence of economically disadvantaged populations ensnared by dynamics of *poverty traps*, as the city’s poverty rate (11.80%) [24] exceeds the national average. A study defined these traps as self-reinforcing cycles of social and economic conditions that keep communities in persistent poverty, discouraging migration to areas with better opportunities [46]. A key factor is present mindedness, a form of steep future discounting identified as both a cause and effect of low socioeconomic status [47]. Such a mindset prioritizes immediate gratification (like low-wage work) over long-term investments like education, reinforcing poverty over a lifetime and across generations [47].

The Townsend deprivation index (TDI) is a composite measure reflecting multiple socioeconomic factors, such as non-homeownership, lack of vehicle access, unemployment, and household overcrowding [18]. GWR coefficient map showed that lower deprivation was associated with lower stroke prevalence across almost all census tracts in the study area. This finding corroborates research by Sheth and colleagues who found that residents of the most deprived neighborhoods were more likely to have co-morbid conditions and experienced higher rates of adverse cardiovascular events, including stroke [48].

Geographically weighted regression (GWR) analysis revealed that better food access, defined as proximity to grocery stores and supermarkets, was associated with lower stroke prevalence. This pattern was consistent across the region, except for a few census tracts in northern Pennsylvania. According to Ogojiaku and colleagues, low food access is defined by living more than 0.5 or 1 mile from a grocery store or supermarket in an urban area, or more than 10 miles in a rural area [18]. The availability of healthier food options is greater in supermarkets than in convenience stores, and neighborhoods with lower socioeconomic status tend to have less healthy food environments, with more fast-food and unhealthy options [49]. Our findings are consistent with another study that reported a negative association between dietary treatment and stroke prevalence, suggesting that dietary interventions can reduce stroke risk [50].

The job participation index, representing the percentage of individuals aged 16–64 in the active labor force [18] was inversely associated with stroke prevalence across most census tracts in HHS Region 3, as shown by the GWR coefficient map. This finding is consistent with a similar study that established a positive correlation between labor force participation and the physical and mental well-being of older adults [51]. They attributed these benefits, in part, to the physical exercise derived from work, suggesting it could help prevent stroke-related chronic conditions like hypertension and diabetes [51].

In contrast, some tracts of Centre County, Pennsylvania, showed that decreased job participation was linked to a lower prevalence of stroke. This finding can be attributed to the large student population in the town of State College, where Penn State University is located. Most of these students are younger, unemployed, and at a lower risk for stroke compared to the general population. Some researchers have highlighted that higher educational attainment often delays entry into the workforce, which accounts for the reduced labor force participation among young adults [52]. Given that stroke risk is known to increase with age [53], the concentration of a younger, pre-labor force population provides a plausible explanation for the reduced stroke prevalence in this locale. The analysis demonstrates a localized trend driven by the area’s specific demographics, and should be considered alongside the broader state context, as Pennsylvania’s overall labor force participation rate (62.80%) is below the national average [54].

Spatial segregation indicator measures the evenness of racial distributions across a spatial unit [55]. Findings from our GWR coefficient map indicate that the relationship between population diversity and stroke prevalence varies geographically. In Allegheny County, located in western Pennsylvania and encompassing Pittsburgh and its suburbs, an increase in diversity was associated with a decrease in stroke prevalence. Conversely, statistically significant tracts in West Virginia, the least diverse state in HHS Region 3 (90.90% White) [45], showed that decreased diversity was associated with increased stroke prevalence.

A study highlighted racial residential segregation as a fundamental mechanism of institutional racism contributing to health disparities in the United States [55]. Their study reported that segregation was associated with a 12% increased hazard of cardiovascular disease, such as stroke, among Black adults [55]. The mechanism by which segregation affects health is multifaceted. As a precursor to community poverty, it systematically limits access to vital resources, including quality education, employment, healthcare, healthy food, and safe spaces for physical activity [55]. This link between residential segregation and CVD incidence in Black adults is also supported by Sims and colleagues [56].

Studies indicate that providing access to healthcare does not always translate into its utilization by the population. Our GWR map of census tracts in Wards 4, 5, 7, and 8 of DC, along with areas in Baltimore and Philadelphia, illustrates this point, revealing a positive association between greater healthcare access and higher stroke prevalence.

This paradoxical finding reinforces the observation that factors beyond insurance coverage affect healthcare access, a reality reflected in DC’s ongoing health outcome disparities for African American residents, despite the city’s near-universal insurance coverage (97%) [37,57,58].

Healthcare disparities in Washington, D.C., are driven by a range of complex issues, including gentrification, demographic changes, and distrust between patients and healthcare providers [37]. Financial and logistical barriers, such as copayments, out-of-pocket costs, and transportation challenges also limit access to care [37]. Furthermore, linguistic diversity, with over 168 languages spoken in the area, along with cultural and educational factors, contributes to inequities and underutilization of services [59]. Other barriers include a general distrust of the medical system and limited telemedicine options [59]. The issue is regional, as the D.C., Baltimore, and Philadelphia corridor is home to an elevated proportion of vulnerable people.

Income Inequality GWR Coefficient map showed that as income gap widened (measured by Gini index), stroke prevalence increased in census tracts in DC. In 2016, D.C. recorded the highest Gini coefficient for income inequality in the nation (0.542), outranking Maryland and Virginia [60]. This extreme disparity, particularly between Black and white residents as noted by the DC Policy Center, is also linked to higher poverty levels in the district [37,60], which may collectively account for the elevated stroke prevalence demonstrated in Appendix L. Conversely, as income gap diminished in census tracts in Prince Georgia County, stroke prevalence increased.

A study reported greater income inequality and a lower standard of living in the southern and midwestern United States compared to other geographic regions [61]. Other researchers investigated the clinical impact of neighborhood income inequality on recovery from ischemic stroke [62]. Their analysis demonstrated that greater neighborhood income inequality, as measured by the GINI Index, is associated with a reduced likelihood of functional independence following hospitalization [62]. Specifically, patients residing in the highest inequality neighborhoods were 250% less likely to achieve independence than those in the lowest inequality neighborhoods [62].

GWR coefficient map for environmental hazard indicator showed negative coefficients in census tracts in Norfolk, Portsmouth, and Danville areas in Virginia. Hence, as environmental pollution increased (less friendly environmental conditions), stroke prevalence increased. Norfolk’s environmental issues, stemming from shipyard and Superfund sites, present potential health risks, including a documented link to stroke [63]. Multiple studies have demonstrated a connection between environmental exposure and stroke. For instance, a study found that even short-term exposure to PM2.5 at concentrations below Israeli air quality guidelines was associated with increased stroke risk [64]. Other research points to specific pollutants, one of such studies linked ozone (O3) concentration to emergency stroke cases, especially in men under 60 and those with pre-existing hypertension [65]. Further, an ecological study across seven southern U.S. states indicated that residential proximity to petroleum production and refining was significantly associated with higher stroke prevalence due to related sulfur dioxide exposure [66].

Conversely, positive coefficients in census tracts around the city of Philadelphia show that, as environmentally friendly conditions increase, stroke prevalence increased. This unexpected finding was observed despite the city’s implementation of several green initiatives, including a plastic bag ban in 2021, the purchase of electric school buses, the operation of a mobile monitoring unit for toxins, and a reported decrease in poor air quality days [67]. Furthermore, Philadelphia’s airport has achieved carbon accreditation, demonstrating a commitment to environmental improvements [67]. The apparent contradiction between improving environmental conditions and rising stroke rates may be influenced by confounding demographic factors. Specifically, with 20.4% of the population aged 60 or older [54], the city has a large, high-risk population for stroke [53], which could account for the observed increase.

### 4.1. Policy Implications

Our findings provide a critical foundation for policy decisions related to community development, urban planning, zoning, housing, transportation, etc. By demonstrating how the neighborhood context and built environment influence stroke prevalence, this research can inform coordinated public health interventions that reduce health disparities. Ultimately, shaping the environment can facilitate positive changes in modifiable stroke risk factors and drive broader social progress. Specific implications of this study are discussed in the subsequent paragraphs.

Our study in HHS Region 3 identifies employment access as a modifiable SDOH affecting stroke prevalence. We found that the link between employment and stroke prevalence varies significantly by neighborhood, allowing for precise, location-specific policy interventions. These findings can guide government and private entities in prioritizing areas for targeted support, such as transportation, childcare, and job training programs. We recommend offering incentives to employers who hire and retain vulnerable residents, as well as developing policies to foster local entrepreneurship [33].

We found a divergent food access-stroke prevalence association, which differed significantly from one Census tract to another in HHS Region 3. This geographical understanding is fundamental for designing policy interventions that effectively address food access limitations and target food deserts. Such interventions should focus on increasing grocery store access, improving transportation, and reducing poverty, which are key drivers of food access issues [37]. Our findings offer vital evidence for local and state-level public health and government initiatives aimed at tackling food deserts in HHS Region 3 [68].

Our geospatial study underscores the need for policymakers to address factors that drive neighborhood segregation. Since racial segregation influences neighborhood socioeconomic opportunities, policies that alter a neighborhood’s socioeconomic composition can have a lasting influence on its future trajectory [69]. Strategic interventions, such as building government-funded affordable housing in expensive areas or offering housing vouchers for high-opportunity neighborhoods, can foster more inclusive communities [37]. Such initiatives designed to increase “opportunity moves” directly address SDOH, as captured by the HOI, and ultimately enhance public health outcomes, including the reduction in stroke prevalence.

Our findings indicate that the relationship between Townsend deprivation index and stroke prevalence is not uniform within HHS Region 3 but varies across census tracts. This geographical variation points to the need for localized-targeted policy interventions to mitigate deprivation. Such interventions could include home purchase assistance programs (HPAP) for low- and middle-income residents, more flexible credit requirements for low-credit-score individuals, material assistance for families, discounted transit passes and tax incentives for low-income housing developers [70].

The strength and direction of the relationship between affordability index and stroke prevalence differ markedly by census tract in HHS Region 3. According to a 2020 Philadelphia study, housing insecurity is linked to a higher risk of hypertension, a precursor to stroke [56]. Public policy interventions addressing housing and transportation costs—key factors in the HOI affordability index—could increase access to affordable public housing, reduce housing inequality, foster more inclusive neighborhoods [37], and ultimately serve to lower the prevalence of stroke.

Given that transportation is an essential SDOH and influences access to healthcare and healthy food, these findings suggest a clear path for policy intervention. For instance, implementing equitable bike-share systems with discounted memberships for qualifying low-income households could mitigate transportation challenges. Addressing transportation limitations in vulnerable communities is expected to positively impact stroke prevalence across the region [37].

This study represents a notable contribution to the integration of a highly technical GIS-based analysis (geographic weighted regression models) in elucidating the relationships between SDOH and stroke prevalence at the census tract level across five states and one district in HHS Region 3. By adopting a GIS-based approach, our study results illustrate spatial health disparities (related to stroke prevalence) through maps that show variation across census tracts in HHS Region 3.

### 4.2. Limitations of the Study

The HOI is tailored to individual jurisdictions (e.g., states and counties) based on available local datasets and indicators relevant to public health stakeholders. Therefore, the HOI computed for this study (which covers five states and one district in HHS Region 3) differs from indices calculated elsewhere. As a result, direct comparisons of the HOI across different jurisdictions or studies is challenging [18].

Interpreting our findings requires acknowledging the ecological fallacy. The observed relationships between variables at the population level is not guaranteed to manifest at the individual level, a limitation inherent to ecological study designs [9].

Lastly, there are concerns about the generalizability of study findings beyond the 8021 census tracts studied in the five states and one district across HHS Region 3. Nevertheless, this study contributes to the body of knowledge on the social determinants of health and their influence on stroke prevalence.

## 5. Conclusions

The burden of stroke in the United States continues to rise, with prevalence increasing over time and remaining a major contributor to mortality, morbidity, and economic strain. Stroke risk is shaped by a complex interplay of individual and structural factors, including social, economic, physical, and neighborhood-level influences. Many of these determinants are modifiable and represent key targets for public health interventions. Although prior research has focused on individual risk factors to explain disparities in stroke outcomes, our study is the first to operationalize the HOI via a geospatial approach to explore the relationship between neighborhood-level SDOH and stroke prevalence across census tracts in HHS Region 3. This research is crucial for understanding the impact of neighborhood context on stroke prevalence and for developing population-level prevention and management strategies adaptable to diverse community settings.

The present study examined how geospatial variations in stroke prevalence relate to neighborhood-level SDOH across census tracts in Delaware, Maryland, Pennsylvania, Virginia, West Virginia, and the District of Columbia. A composite Health Opportunity Index (HOI) was first calculated for each census tract. Geographically Weighted Regression (GWR) was then performed to map the direction and strength of the relationship between HOI indicators and stroke prevalence. The results provide spatial insights into these associations, supporting the development of more effective, location-specific strategies to address health disparities.

## Figures and Tables

**Table 1:** Estimates of Demographic Characteristics of HHS Region 3 States.

Variables	U.S.	DE	VA	WV	PA	MD	DC
Persons 65+ years	17.70%	21.30%	17.20%	21.50%	20.00%	17.30%	13.10%
Black	13.70%	24.10%	20.00%	3.80%	12.30%	31.60%	44.40%
American Indian/ Alaskan Native	1.30%	0.70%	0.60%	0.30%	0.50%	0.80%	0.70%
Asian	6.40%	4.40%	7.40%	0.90%	4.20%	7.10%	4.90%
Native Hawaiian and other Pacific Islander	0.30%	0.10%	0.10%	0	0.10%	0.10%	0.20%
Two or more races	3.10%	3.10%	3.50%	2.10%	2.40%	3.30%	3.30%
Hispanic or Latino	19.50%	11.10%	11.20%	2.20%	8.90%	12.60%	12.00%
White	58.40%	58.90%	59.10%	90.90%	74.10%	47.30%	37.70%
High school or higher	89.10%	91.20%	91.10%	88.40%	91.70%	91.00%	92.70%
Uninsured (under 65)	9.50%	6.90%	7.60%	7.40%	6.50%	7.10%	3.30%
Civilian labor force (age 16+)	63.00%	61.90%	63.80%	53.10%	62.80%	66.60%	71.40%
Median income	$75,149	$79,325	$87,249	$55,217	$73,170	$98,461	$101,722
Persons in poverty	11.10%	9.40%	10.60%	17.90%	11.80%	9.60%	13.30%

Source: U.S. Census Bureau, 2018–2022 (Quick Facts).

**Table 2:** GWR Model: Association of Neighborhood and Built Environment Profile and Stroke Prevalence in HHS Region 3.

Statistics	GWR
*R*-squared	76.90%
Adjusted *R*-squared	70.42%
AICc	15,537.40
Sigma-squared	0.29
Sigma-squared MLE	0.23
Effective degree of freedom	6252.18

**Table 3:** GWR Model: Association of Social and Community Context Profile and Stroke Prevalence in HHS Region 3.

Statistics	GWR
*R*-squared	79.70%
Adjusted *R*-squared	76.92%
AICc	12,173.69
Sigma-squared	0.23
Sigma-squared MLE	0.2
Effective degree of freedom	7054.00

**Table 4:** GWR Model: Association of Resource Profile and Stroke Prevalence in HHS Region 3.

Statistics	GWR
*R*-squared	68.24%
Adjusted *R*-squared	63.68%
AICc	15,870.75
Sigma-squared	0.36
Sigma-squared MLE	0.32
Effective degree of freedom	7013.40

**Table 5:** GWR Model: Association of Economic Profile and Stroke Prevalence in HHS Region 3.

Statistics	GWR
*R*-squared	69.91%
Adjusted *R*-squared	64.55%
AICc	15,988.29
Sigma-squared	0.35
Sigma-squared MLE	0.3
Effective degree of freedom	6809.00

## Data Availability

The raw data supporting the conclusions of this article will be made available by the authors on request.

## Supplementary Materials

The following supporting information can be downloaded at: https://www.mdpi.com/article/10.3390/ijerph22101542/s1, Supplementary File S1: Profiles 1–4.

## Appendix A. Employment Access Indicator

(1) Positive local coefficients (in green): Positive strengths of association between the HOI indicator and stroke prevalence across CTs. (2) Negative local coefficients (in red): Negative strengths of association between the HOI indicator and stroke prevalence across CTs). (3) Areas shaded deep ash, lighter ash or white represent CTs across the study area where the local parameter estimates are significant at the 0.05 alpha level, 0.1 alpha level or non-significant (white), respectively. (4) Blown-out map shows clustered CTs.

## Appendix B. Affordability Indicator

(1) Positive local coefficients (in green): Positive strengths of association between the HOI indicator and stroke prevalence across CTs. (2) Negative local coefficients (in red): Negative strengths of association between the HOI indicator and stroke prevalence across CTs). (3) Areas shaded deep ash, lighter ash or white represent CTs across the study area where the local parameter estimates are significant at the 0.05 alpha level, 0.1 alpha level or non-significant (white), respectively. (4) Blown-out map shows clustered CTs.

## Appendix C. Walkability Indicator

(1) Positive local coefficients (in green): Positive strengths of association between the HOI indicator and stroke prevalence across CTs. (2) Negative local coefficients (in red): Negative strengths of association between the HOI indicator and stroke prevalence across CTs). (3) Areas shaded deep ash, lighter ash or white represent CTs across the study area where the local parameter estimates are significant at the 0.05 alpha level, 0.1 alpha level or non-significant (white), respectively. (4) Blown-out map shows clustered CTs.

## Appendix D. Population Weighted Density Indicator

(1) Positive local coefficients (in green): Positive strengths of association between the HOI indicator and stroke prevalence across CTs. (2) Negative local coefficients (in red): Negative strengths of association between the HOI indicator and stroke prevalence across CTs). (3) Areas shaded deep ash, lighter ash or white represent CTs across the study area where the local parameter estimates are significant at the 0.05 alpha level, 0.1 alpha level or non-significant (white), respectively. (4) Blown-out map shows clustered CTs.

## Appendix E. Geographic Mobility Indicator

(1) Positive local coefficients (in green): Positive strengths of association between the HOI indicator and stroke prevalence across CTs. (2) Negative local coefficients (in red): Negative strengths of association between the HOI indicator and stroke prevalence across CTs). (3) Areas shaded deep ash, lighter ash or white represent CTs across the study area where the local parameter estimates are significant at the 0.05 alpha level, 0.1 alpha level or non-significant (white), respectively. (4) Blown-out map shows clustered CTs.

## Appendix F. Townsend Deprivation Indicator

(1) Positive local coefficients (in green): Positive strengths of association between the HOI indicator and stroke prevalence across CTs. (2) Negative local coefficients (in red): Negative strengths of association between the HOI indicator and stroke prevalence across CTs). (3) Areas shaded deep ash, lighter ash or white represent CTs across the study area where the local parameter estimates are significant at the 0.05 alpha level, 0.1 alpha level or non-significant (white), respectively. (4) Blown-out map shows clustered CTs.

## Appendix G. Food Access Indicator

(1) Positive local coefficients (in green): Positive strengths of association between the HOI indicator and stroke prevalence across CTs. (2) Negative local coefficients (in red): Negative strengths of association between the HOI indicator and stroke prevalence across CTs). (3) Areas shaded deep ash, lighter ash or white represent CTs across the study area where the local parameter estimates are significant at the 0.05 alpha level, 0.1 alpha level or non-significant (white), respectively. (4) Blown-out map shows clustered CTs.

## Appendix H. Education Indicator

(1) Positive local coefficients (in green): Positive strengths of association between the HOI indicator and stroke prevalence across CTs. (2) Negative local coefficients (in red): Negative strengths of association between the HOI indicator and stroke prevalence across CTs). (3) Areas shaded deep ash, lighter ash or white represent CTs across the study area where the local parameter estimates are significant at the 0.05 alpha level, 0.1 alpha level or non-significant (white), respectively. (4) Blown-out map shows clustered CTs.

## Appendix I. Job Participation Indicator

(1) Positive local coefficients (in green): Positive strengths of association between the HOI indicator and stroke prevalence across CTs. (2) Negative local coefficients (in red): Negative strengths of association between the HOI indicator and stroke prevalence across CTs). (3) Areas shaded deep ash, lighter ash or white represent CTs across the study area where the local parameter estimates are significant at the 0.05 alpha level, 0.1 alpha level or non-significant (white), respectively. (4) Blown-out map shows clustered CTs.

## Appendix J. Segregation Indicator

(1) Positive local coefficients (in green): Positive strengths of association between the HOI indicator and stroke prevalence across CTs. (2) Negative local coefficients (in red): Negative strengths of association between the HOI indicator and stroke prevalence across CTs). (3) Areas shaded deep ash, lighter ash or white represent CTs across the study area where the local parameter estimates are significant at the 0.05 alpha level, 0.1 alpha level or non-significant (white), respectively. (4) Blown-out map shows clustered CTs.

## Appendix K. Health Access Indicator

(1) Positive local coefficients (in green): Positive strengths of association between the HOI indicator and stroke prevalence across CTs. (2) Negative local coefficients (in red): Negative strengths of association between the HOI indicator and stroke prevalence across CTs). (3) Areas shaded deep ash, lighter ash or white represent CTs across the study area where the local parameter estimates are significant at the 0.05 alpha level, 0.1 alpha level or non-significant (white), respectively. (4) Blown-out map shows clustered CTs.

## Appendix L. Income Inequality Indicator

(1) Positive local coefficients (in green): Positive strengths of association between the HOI indicator and stroke prevalence across CTs. (2) Negative local coefficients (in red): Negative strengths of association between the HOI indicator and stroke prevalence across CTs). (3) Areas shaded deep ash, lighter ash or white represent CTs across the study area where the local parameter estimates are significant at the 0.05 alpha level, 0.1 alpha level or non-significant (white), respectively. (4) Blown-out map shows clustered CTs.

## Appendix M. Environmental Hazard Indicator

(1) Positive local coefficients (in green): Positive strengths of association between the HOI indicator and stroke prevalence across CTs. (2) Negative local coefficients (in red): Negative strengths of association between the HOI indicator and stroke prevalence across CTs). (3) Areas shaded deep ash, lighter ash or white represent CTs across the study area where the local parameter estimates are significant at the 0.05 alpha level, 0.1 alpha level or non-significant (white), respectively. (4) Blown-out map shows clustered CTs.
