# Supplementary material for: A Geographic Weighted Regression Analysis of the Health Opportunity Index and Stroke Prevalence in Health and Human Services Region 3
Source: Int J Environ Res Public Health. 2025 Oct 9;22(10):1542. doi: 10.3390/ijerph22101542 (PMC12562347; doi:10.3390/ijerph22101542)
Supplement: Supplementary file 1 [file ijerph-22-01542-s001.zip › ijerph-3776795-supplementary.pdf]

Profile 1

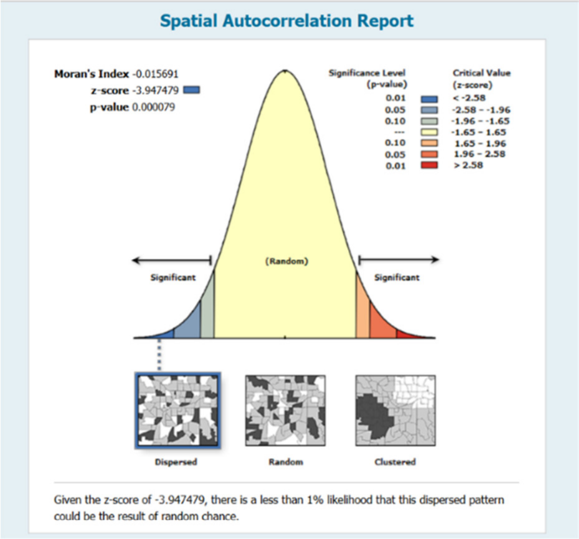

Global Moran's I Summary

**Moran's Index**	-0.015691
**Expected Index**	-0.000125
**Variance**	0.000016
**z-score**	-3.947479
**p-value**	0.000079

Dataset Information

**Input Feature Class:**	PROFILE 1 GROUP\WALKABILITY_C
**Input Field:**	S_STDRESID
**Conceptualization:**	INVERSE_DISTANCE_SQUARED
**Distance Method:**	EUCLIDEAN
**Row Standardization:**	True
**Distance Threshold:**	40797.0000 meters
**Weights Matrix File:**	None
**Selection Set:**	False

Global Moran's I Summary

Moran's Index	-0.015691
Expected Index	-0.000125
Variance	0.000016
z-score	-3.947479
p-value	0.000079

Distance measured in meters
Writing html report....
C:\Users\tukturw\OneDrive - Government of The District of Columbia\Documents\PERSONAL FILES\DISERTATION\NEW ANALYSIS_08_21_24\MGWR_PROFILE1\PROFILE1_08_21_2024\MoransI_Result_34508_33028_.html
Succeeded at Sunday, September 14, 2025 5:45:14 PM (Elapsed Time: 18.47 seconds)

Profile 2

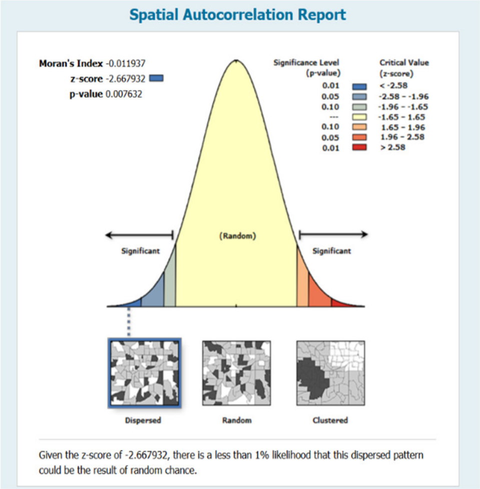

Global Moran's I Summary

**Moran's Index**	-0.011937
**Expected Index**	-0.000125
**Variance**	0.000020
**z-score**	-2.667932
**p-value**	0.007632

Dataset Information

**Input Feature Class:**	PROFILE 2\TOWNSEND_T
**Input Field:**	S_STDRESID
**Conceptualization:**	INVERSE_DISTANCE_SQUARED
**Distance Method:**	EUCLIDEAN
**Row Standardization:**	True
**Distance Threshold:**	25384.0000 meters
**Weights Matrix File:**	None
**Selection Set:**	False

Global Moran's I Summary

Moran's Index	-0.011937
Expected Index	-0.000125
Variance	0.000020
z-score	-2.667932
p-value	0.007632

Distance measured in meters
Writing html report....
C:\Users\tukturw\OneDrive - Government of The District of Columbia\Documents\PERSONAL FILES\DISERTATION\NEW ANALYSIS_08_21_24\MGWR_PROFILE2\PROFILE2_08_21_2024\MoransI_Result_36352_5828_.html
Succeeded at Sunday, September 14, 2025 6:11:21 PM (Elapsed Time: 14.21 seconds)

Profile 3

Spatial Autocorrelation Report

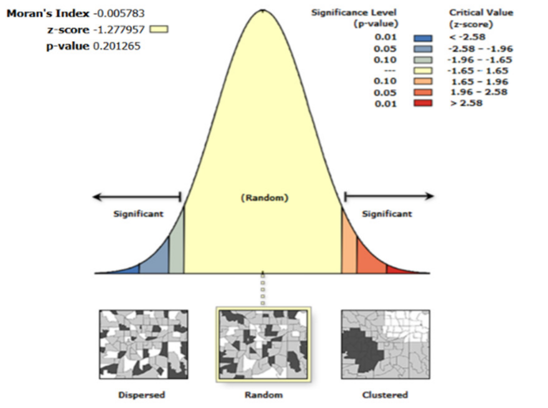

Given the z-score of -1.277957, the pattern does not appear to be significantly different than random.

Global Moran's I Summary

**Moran's Index**	-0.005783
**Expected Index**	-0.000125
**Variance**	0.000020
**z-score**	-1.277957
**p-value**	0.201265

Dataset Information

**Input Feature Class:**	PROFILE 3\SEGREGATION_T
**Input Field:**	S_STDRESID
**Conceptualization:**	INVERSE_DISTANCE_SQUARED
**Distance Method:**	EUCLIDEAN
**Row Standardization:**	True
**Distance Threshold:**	25383.3692 meters
**Weights Matrix File:**	None
**Selection Set:**	False

Global Moran's I Summary

Moran's Index	-0.005783
Expected Index	-0.000125
Variance	0.000020
z-score	-1.277957
p-value	0.201265

Distance measured in meters
Writing html report....
C:\Users\tukturw\OneDrive - Government of The District of Columbia\Documents\PERSONAL FILES\DISERTATION\NEW ANALYSIS_08_21_24\MGWR_PROFILE_3\PROFILE3_08_21_2024\MoransI_Result_32176_18068_.html
Succeeded at Sunday, September 14, 2025 6:18:27 PM (Elapsed Time: 14.10 seconds)

Profile 4

Spatial Autocorrelation Report

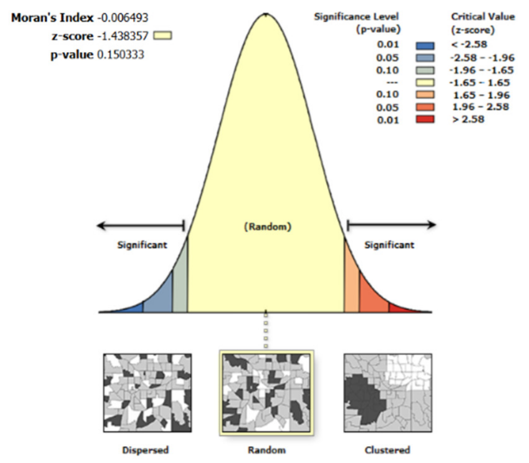

Given the z-score of -1.438357, the pattern does not appear to be significantly different than random.

Global Moran's I Summary

**Moran's Index**	-0.006493
**Expected Index**	-0.000125
**Variance**	0.000020
**z-score**	-1.438357
**p-value**	0.150333

Dataset Information

**Input Feature Class:**	PROFILE 4\INCOME INEQUALITY_C
**Input Field:**	S_STDRESID
**Conceptualization:**	INVERSE_DISTANCE_SQUARED
**Distance Method:**	EUCLIDEAN
**Row Standardization:**	True
**Distance Threshold:**	25383.3692 meters
**Weights Matrix File:**	None
**Selection Set:**	False

Global Moran's I Summary

Moran's Index	-0.006493
Expected Index	-0.000125
Variance	0.000020
z-score	-1.438357
p-value	0.150333

Distance measured in meters
Writing html report....
C:\Users\tukturw\OneDrive - Government of The District of Columbia\Documents\PERSONAL_FILES\DISERTATION\NEW_ANALYSIS_08_21_24\MGWR_PROFILE4\PROFILE4_08_21_2024\MoransI_Result_16148_9228_0.html
Succeeded at Sunday, September 14, 2025 6:30:53 PM (Elapsed Time: 8.94 seconds)
